# Volatile Compounds From *Bacillus*, *Serratia*, and *Pseudomonas* Promote Growth and Alter the Transcriptional Landscape of *Solanum tuberosum* in a Passively Ventilated Growth System

**DOI:** 10.3389/fmicb.2021.628437

**Published:** 2021-07-21

**Authors:** Darren Heenan-Daly, Simone Coughlan, Eileen Dillane, Barbara Doyle Prestwich

**Affiliations:** ^1^School of Biological, Earth and Environmental Sciences, University College Cork, Cork, Ireland; ^2^Environmental Research Institute, University College Cork, Cork, Ireland; ^3^School of Mathematics, Statistics and Applied Mathematics, National University of Ireland, Galway, Ireland

**Keywords:** volatile organic compounds, plant-bacteria interactions, plant growth-promotion, biocontrol, GC/MS, transcriptomics

## Abstract

The interaction of an array of volatile organic compounds (VOCs) termed bacterial volatile compounds (BVCs) with plants is now a major area of study under the umbrella of plant-microbe interactions. Many growth systems have been developed to determine the nature of these interactions *in vitro*. However, each of these systems have their benefits and drawbacks with respect to one another and can greatly influence the end-point interpretation of the BVC effect on plant physiology. To address the need for novel growth systems in BVC-plant interactions, our study investigated the use of a passively ventilated growth system, made possible via Microbox^®^ growth chambers, to determine the effect of BVCs emitted by six bacterial isolates from the genera *Bacillus*, *Serratia*, and *Pseudomonas*. Solid-phase microextraction GC/MS was utilized to determine the BVC profile of each bacterial isolate when cultured in three different growth media each with varying carbon content. 66 BVCs were identified in total, with alcohols and alkanes being the most abundant. When cultured in tryptic soy broth, all six isolates were capable of producing 2,5-dimethylpyrazine, however BVC emission associated with this media were deemed to have negative effects on plant growth. The two remaining media types, namely Methyl Red-Voges Proskeur (MR-VP) and Murashige and Skoog (M + S), were selected for bacterial growth in co-cultivation experiments with *Solanum tuberosum* L. cv. ‘Golden Wonder.’ The BVC emissions of *Bacillus* and *Serratia* isolates cultured on MR-VP induced alterations in the transcriptional landscape of potato across all treatments with 956 significantly differentially expressed genes. This study has yielded interesting results which indicate that BVCs may not always broadly upregulate expression of defense genes and this may be due to choice of plant-bacteria co-cultivation apparatus, bacterial growth media and/or strain, or likely, a complex interaction between these factors. The multifactorial complexities of observed effects of BVCs on target organisms, while intensely studied in recent years, need to be further elucidated before the translation of lab to open-field applications can be fully realized.

## Introduction

The relationship between plants and their consortia of microbial associates is recognized as one of the most ancient relationships on Earth. Today, we have the opportunity to exploit this relationship which can benefit the environment and thus, society as a whole. Numerous studies have demonstrated the ability of plant-associated bacteria, particularly rhizobacteria which are found in and around the root zone of the plant, to aid plant development and growth (Bloemberg and Lugtenberg, 2001; Adesemoye et al., 2009; Lugtenberg and Kamilova, 2009; Vejan et al., 2016; Méndez-Bravo et al., 2018; Basu et al., 2021). In the natural environment, rhizobacteria employ both direct and indirect plant growth-promoting (PGP) activities to optimize the health and growth of their plant hosts (Ghyselinck et al., 2013). Direct PGP activities include the sequestration of iron through the production of siderophores (Ghosh et al., 2020), the solubilization of phosphorus (Adnan et al., 2020), alteration of phytohormone levels such as IAA (Raut et al., 2017) and the modulation of ethylene levels within the host (Nascimento et al., 2018). Indirect mechanisms can include the secretion of lytic enzymes (Jadhav et al., 2017), competition for nutrients (Haggag and Timmusk, 2008), the induction of systemic resistance (Jacob et al., 2020) and the emission of volatile organic compounds (Fincheira et al., 2021). The latter two of these activities can often work synergistically where volatiles alone can induce systemic resistance as demonstrated in *Arabidopsis thaliana* (Ryu et al., 2004). Volatile compounds, particularly volatile organic compounds, often serve as chemical communication signals in both intra- and interspecific communication between kingdoms (Schulz-Bohm et al., 2017). Of the numerous activities that rhizobacteria employ to aid plant growth, BVCs have emerged as a key area of investigation over the last two decades (Ryu et al., 2003; Farag et al., 2006, 2017) and their future application to agricultural production systems are an attractive option, especially with regard to sustainable agriculture (Heenan-Daly et al., 2019; Garbeva and Weisskopf, 2020).

The fact that these chemical signals are naturally produced by many rhizobacterial species presents an opportunity for application in the field as part of a sustainable approach to agricultural production systems. Application of the rhizobacterial isolates themselves to release potentially beneficial BVCs or indeed the application of pure volatile compounds may help to reduce the application of environmentally harmful biocides and fertilizers, and numerous studies have addressed how this could be implemented in the field, with particular interest in overcoming problems with high volatility such as applying volatiles as part of oil-in-water multilayer emulsions (Choi et al., 2014; Fincheira et al., 2019, 2020). BVCs can act in two ways to promote plant growth; firstly, they can directly inhibit the growth of fungal and bacterial phytopathogens so as to prevent them from damaging or killing a potential host (Vespermann et al., 2007; Asari et al., 2016). Secondly, they can act to modulate gene expression within the sensor plant perceiving the volatile signal, often involving genes involved in growth, defense and nutrient acquisition (Gutiérrez-Luna et al., 2010; Farag et al., 2013; Zamioudis et al., 2015; Hernández-Calderón et al., 2018; Romera et al., 2019). It is widely reported that during *in vitro* experimentation the substrate on which the bacterial isolate proliferates and its own particular capacity for volatile emission can affect the composition of its corresponding VOC profile, having both positive and negative effects on growth, for example different *Pseudomonas* strains were shown to emit varying amounts of hydrogen cyanide when grown on Luria Broth (LB) agar and this could lead to severely deleterious phytotoxic effects in *A. thaliana* when excessive emission occurred (>17 μmol), in fact when compared to other media such as nutrient rich MR-VP or the less nutrient rich Angle and M + S, LB media was the only one to show plant lethality (Blom et al., 2011a, b). This can have implications for the effect of BVCs applied both in the lab and in the field with conditions such as temperature, humidity and nutrient availability affecting the emission and volatilization of these compounds and thus, potentially, their respective effect on growth (Chung et al., 2016). The effect of BVCs on modulation of gene expression related to defense, growth and nutrient acquisition has been demonstrated in numerous studies since the publications of Ryu et al. (2003) and Ryu et al. (2004) regarding the volatile-mediated regulation of gene expression in *A. thaliana*. Indeed, this activity, rather than the direct inhibition of phytopathogens, has become a more attractive option for improved agricultural practices as BVCs can “prime” the target plant by activation of an induced systemic resistance (ISR) (Choi et al., 2014).

In the lab, closed systems have been the popular choice for observing the experimental effects of VOCs on target organisms due to their relative ease-of-use and affordability. The most common approach is the use of split-plate Petri dishes, often referred to as ‘I plates,’ where VOCs can diffuse from one chamber of the plate to the other over a central dividing septum (Velivelli et al., 2015). Closed systems have a number of drawbacks, however, the first being that the build-up and subsequent increased concentration of VOCs within these systems is highly unlikely to occur in an open system or especially in the field. The second is that given the right conditions, the effect of VOCs can be detrimental, or even lethal, to the target organism (Blom et al., 2011b), as we observed in plants that were exposed to BVCs from TSB-cultured isolates. Open systems represent a more true-to-life effect of VOCs on target organisms (Kai and Piechulla, 2009) but have the drawback of laborious mechanical apparatus such as pumps and the added expense which these can bring. This study investigates the effect of BVCs on plant growth and defense in *S. tuberosum* L. cv. ‘Golden Wonder’ utilizing a novel co-cultivation method, and solid phase micro-extraction gas chromatography/mass spectrometry (SPME-GC/MS). Our approach was to find a “middle ground,” where the outer and inner atmosphere of the system could passively interact and exchange between one another to avoid an excessive accumulation of bacterial and indeed plant-derived volatiles, while still maintaining an axenic environment within the system thanks to the Microbox^®^ growth chamber, along with providing relative ease-of-use and affordability. Volatiles affect all areas of plant morphology both above- and belowground (Wenke et al., 2010; Sharifi and Ryu, 2018) and therefore it is prudent to allow BVC blends to potentially access all areas of the plant during experimental analyses to determine the systemic effect on plant growth and/or gene expression. To address this, polyurethane (PE) foam was selected in which to grow potato microplants for this study as it represented a more neutral substrate in which to cultivate the plant compared to sterilized soil, which has been used in some closed systems (Park et al., 2015; Tahir et al., 2017). The additional advantage of using PE foam is that, like soil, the foam is a porous matrix thus allowing potential diffusion of BVCs to the root system. Taken together, the use of the passively ventilated system investigated here may serve to negate any potential effects (positive and/or negative) of the over-accumulation of BVCs within closed *in vitro* growth systems, the use of PE foam may negate any potential nutritive effects of sterilized soil which could impact plant growth rates and also allows plant-wide exposure to BVCs from the shoots to the roots. Additionally, the size of this system allows for the investigation of larger and more mature plants than *A. thaliana* which is often studied in I-plates, the expanded range of plant subjects which can be investigated in this system may help to further elucidate species-specific effects of certain volatiles. Indeed, more than one microbe at a time may be investigated, and the potential inclusion of insects within the system could be employed as part of a wider biocontrol investigation using PGP microbes.

The aim of this study was to investigate the effect of BVCs on plant growth with five rhizobacterial isolates obtained from Irish potato soils growing on two different media types, MR-VP and M + S, in the presence of *S. tuberosum* L. cv. ‘Golden Wonder’ within a passively ventilated growth system. Studies concerning the effect of BVCs on plant growth employ either closed or open growth systems which can affect the experimental outcome and as a consequence, can potentially misrepresent the efficiency of these molecules in any potential agricultural field application. SPME-GC/MS was used in this study to qualitatively determine the volatile profiles of bacterial isolates in three different media types, each with varying levels of carbon content. Transcriptomic analysis using MACE (massive analysis of cDNA ends) RNA-seq allowed identification of differentially expressed genes in *S. tuberosum* in response to BVCs from *Bacillus amyloliquefaciens* FZB24^®^ and *Serratia fonticola* LAC2.

## Materials and Methods

### *Solanum tuberosum* Tissue Culture Maintenance

Microplants of *S. tuberosum* L. cv. ‘Golden Wonder’ were supplied by the TOPS potato centre (Department of Agriculture, Food and Marine, Raphoe, Donegal, Ireland). Stocks were maintained on 1/2 strength Murashige and Skoog (M + S) agar consisting of: 2.2 g basal M + S medium (Sigma Aldrich #5519), 15 g sucrose, 6 g agar (Sigma Aldrich) per liter with final pH adjusted to 5.80. Nodal sections of microplants were subcultured on fresh media under aseptic conditions every 4-5 weeks in Microbox^®^ ‘TP 3000’ growth chambers equipped with ‘XXL + ’ filters (Combiness, Belgium). The microplants were kept in a growth room at 23°C under a 16 h d^–1^ photoperiod and a photosynthetic photon flux (PPF) of 225 μmol m^–1^ s^–1^.

### Bacterial Stock Maintenance

The commercial PGPR strain *Bacillus amyloliquefaciens* FZB24^®^ and the following isolates from Irish potato soils *Serratia fonticola* LAC2, *Pseudomonas azotoformans* BRP14, *Bacillus toyonensis* BMC10, *Bacillus mycoides* LAM7 and *Serratia myotis* LAM10 were used in all experiments. Bacterial isolates were maintained on tryptic soy agar (TSA) (Sigma Aldrich). A single bacterial colony was streaked on TSA in a 90 mm Petri dish (Sarstedt, Germany) and allowed to grow overnight at 28°C ± 2°C or until visible colonies were observed. Cultures were Parafilmed and stored at 4°C for 4-5 weeks or maintained long term at -80°C in a 50% glycerol solution. The 16S rRNA sequences for potato soil isolates are deposited in GenBank under the accession numbers MT373405, MT373394, MT373410, MT373402, and MT373403.

### GC/MS Analysis of Putative BVCs From Selected Rhizobacteria

Solid phase micro-extraction gas chromatography/mass spectrometry analysis was utilized to determine VOC profiles of rhizobacteria according to Velivelli et al. (2015) with slight modifications. SPME fibers were sourced from Supelco (Sigma Aldrich) and conditioned prior to use as per manufacturer’s instructions. Rhizobacterial isolates were grown overnight on plates of TSA and incubated at 28 ± 2°C. A single colony from each isolate was used to inoculate 20 ml glass vials (Sigma Aldrich) containing 9 ml of TSB, MR-VP or M + S under aseptic conditions. The vials were sealed with metal crimp caps (Sigma Aldrich) fitted with rubber septa (Sigma Aldrich) ensuring that they were gastight. All components were autoclaved individually prior to inoculation. Vials were incubated at 28°C ± 2°C for 24–72 h at 170 rpm. Volatile analysis was carried out after 24, 48, and 72 h using a Shimadzu GC/MS-QP5000 equipped with a DB-5 column (30 m × 0.25 mm × 0.25 μm). Sample vials were placed in an incubator at 50°C. The SPME fiber (divinylbenzene/carboxen/polydimethylsiloxane (DCP, 50/30 μm)) was then inserted and exposed to each rhizobacterial BVC sample in the vial headspace for 40 min to allow adsorption of respective volatiles. The fiber containing the volatiles was then desorbed at 210°C for 1 min in the injection port of a gas chromatograph coupled to a mass spectrometer. The GC/MS run time was 22 min, and the injection port was operated in a split mode with a constant He flow of 1.0 ml min^–1^. The initial oven temperature was set at 33°C for 3 min, increased to 180°C at a rate of 10°C min^–1^, further increased to 220°C at a rate of 40°C min^–1^, and with a final hold for 5 min at 220°C. Mass spectra were obtained in the electron ionization (EI) mode at 70 eV, with a continuous *m/z* scan from 40 to 250. The SPME fiber was conditioned after each run for 10 min at 210°C in the injection port and a cleaning run ‘burn-off’ was conducted before the next sample was analyzed. Identification of the resulting VOCs was done by comparison of volatile blend mass spectra with those of the NIST05/21, SZTERP mass spectra library (similarity index > 90%). The experiment was repeated twice per isolate/media combination and media controls were included during bacterial media inoculation.

### Plant/Bacteria Co-cultivation for BVC Exposure

Four nodal sections of 4-week-old stock plants of *S. tuberosum* L. cv. ‘Golden Wonder’ were placed in polyurethane foams (supplied by the School of Biological, Earth and Environmental Sciences, UCC, Ireland) in magenta containers imbibed with 50 ml autotrophic medium (1/2 strength M + S basal medium (Sigma Aldrich #5519) 2.2 g per liter, pH adjusted to 5.80). These planted magentas were placed in ‘TP5000 + TPD5000’ Microbox^®^ growth chambers equipped with ‘XXL + ’ filters (Combiness, Belgium). These growth chambers were placed in a growth room under a 16 h photoperiod at 23°C for 2 weeks to allow the microplants to acclimatize to surrounding environmental conditions. Following this the plantlets were exposed to BVCs by dropping 20 μl of each bacterial culture at OD 600nm = 1.0 on the center of a 50 mm Petri dish containing either Methyl Red-Voges Proskeur (MR-VP) broth (Sigma Aldrich) supplemented with 14 g agar (Sigma Aldrich) per liter; or ½ strength M + S media containing 2.2 g M + S basal medium (Sigma Aldrich #5519), 15 g sucrose and 6 g agar per liter. The bacterial inoculum was allowed to air-dry under aseptic conditions for ∼15 min and the Petri dish was then placed inside the Microbox^®^ growth chamber containing the microplants under aseptic conditions. The microplants were exposed to the resulting rhizobacterial BVCs for a period of 4 weeks. Control microplants were set up with either un-inoculated axenic media or no media at all. Experiment was repeated twice with four technical replicates and each technical replicate consisted of four microplants.

### RNA Extraction

Microplants exposed to BVCs from LAC2 (L), FZB24 (F), or absolute control (C) or control with axenic media (M) were cultivated as described above in an independent experiment. Plants were harvested under aseptic conditions and RNA extractions from stem tissue were carried out using the QIAGEN^®^ RNeasy^TM^ plant extraction kit according to the manufacturer’s specifications. To eliminate genomic DNA contamination the Ambion^®^ TURBO^TM^ DNAse kit was used according to manufacturer’s specifications (Life Technologies, Carlsbad, CA, United States). RNA quantification was measured using a Nanodrop^TM^ 2000c spectrophotometer (Thermo Fisher Scientific, Waltham, MA, United States). Samples were immediately snap frozen in liquid nitrogen and stored at -80°C to prevent degradation of RNA.

### MACE Sequencing

Massive analysis of cDNA (MACE) is a 3′mRNA sequencing method based on the analysis of Illumina reads derived from fragments that originate from 3′ mRNA ends (Müller et al., 2014). The samples were prepared by GenXPro GmbH (Frankfurt, Germany) using the MACE-Kit v1 according to the manual of the manufacturers (GenXPro GmbH). Briefly, RNA was fragmented and polyadenylated mRNA was enriched by poly-A specific reverse transcription. A specific adapter was ligated to the 5′ ends and the 3′ ends were amplified by competitive PCR. Duplicate reads as determined by the implemented unique molecular identifiers (TrueQuant IDs) were removed from the raw dataset. Low quality sequence-bases were removed by the software cutadapt^1^ and poly(A)-tails were clipped by an in-house Python-Script. The RNA-seq data discussed in this publication have been deposited in NCBI’s Gene Expression Omnibus (Edgar et al., 2002) and are accessible through GEO Series accession number GSE160297.

### Statistical and Bioinformatic Analysis

Growth data was statistically analyzed using SPSS 25 (IBM). Two-way analysis of variance (ANOVA) was used to determine the interaction effect of bacterial isolate and media type on plant growth. Significant differences among BVC treatments were estimated using one-way between groups ANOVA with Tukey test for *post hoc* comparisons. For transcriptomic analysis, DeSeq2 version 1.24 was used on raw counts provided by GenXPro, removing genes with no counts or only a single count across all samples prior to analysis. Genes were considered to be differentially expressed if they had a Benjami Hochberg adjusted *p*-Value of (<0.05) and absolute log2 fold change (≥2). Gene Ontology (GO) enrichment was performed using GoSeq version 1.36.0 with significant GO terms identified as those with adjusted *p*-Values (<0.05) for over-represented terms. Gene IDs, descriptions and GO terms were retrieved from Ensembl Biomart v2.40.5 using *Solanum tuberosum* (SolTub_3.0) as the reference. The pheatmap function from the pheatmap v1.0.12 package was used to plot the heat map with clustering distance for rows and columns set to correlation, scale set to row, and row and column clustering set to use complete linkage hierarchical clustering. R version 3.6.1 was used for all analysis. The UpSetR plots were created using UpSetR version 1.4.0 using the gene IDs of genes that were differentially expressed in each comparison.

## Results

### Effect of BVCs on Plant Dry Weight and Stem Length

**FIGURE 1:**
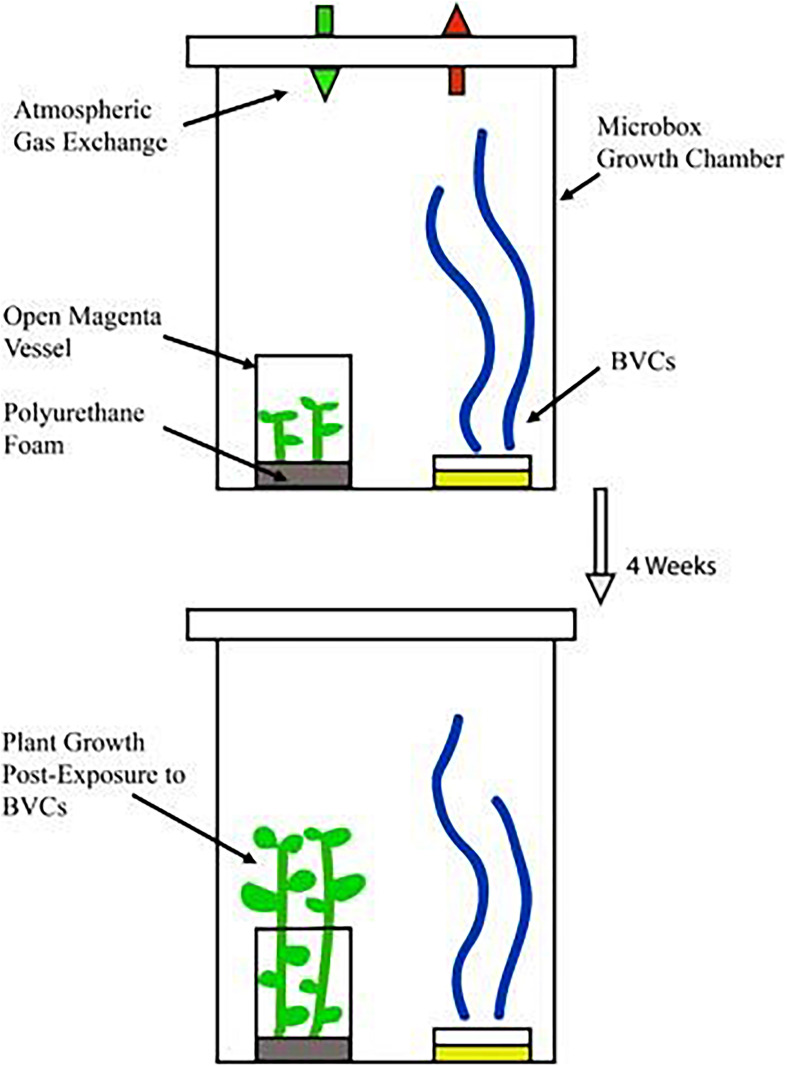
Plants growing in polyurethane foam imbibed with 50 ml M+S media. Bacteria release BVCs from respective growth media and plants are exposed to BVCs within the growth chamber. Gas exchange between the atmosphere and the growth chamber is mediated by the Microbox^®^ growth chamber technology. Plants are co-cultivated with respective physically separated bacteria for 4 weeks.

To test the effect of BVCs from isolates cultured on MR-VP and M + S on plant dry weight and stem length, a 50 mm Petri dish inoculated with each respective isolate, or none in the case of media control treatments, was placed in a TP5000 + TPD5000 Microbox^®^ growth chamber to allow BVCs to diffuse within the chamber thus exposing the plants within the open Magenta vessel to each of the respective isolate BVCs, the inclusion of the XXL + grade allowed maximal passive-ventilation between the growth chamber and outer atmosphere (Figure 1).

Two-way ANOVA was carried out to examine the relationship between bacterial isolate, media type and their interaction effect on plant growth and the resulting dry weight and stem length respectively. The interaction effect of isolate^∗^media on dry weight was not statistically significant (*p* = 0.083) (*p* < 0.01). The interaction effect of isolate^∗^media on stem length was also not statistically significant (*p* = 0.055) (*p* < 0.01).

**FIGURE 2:**
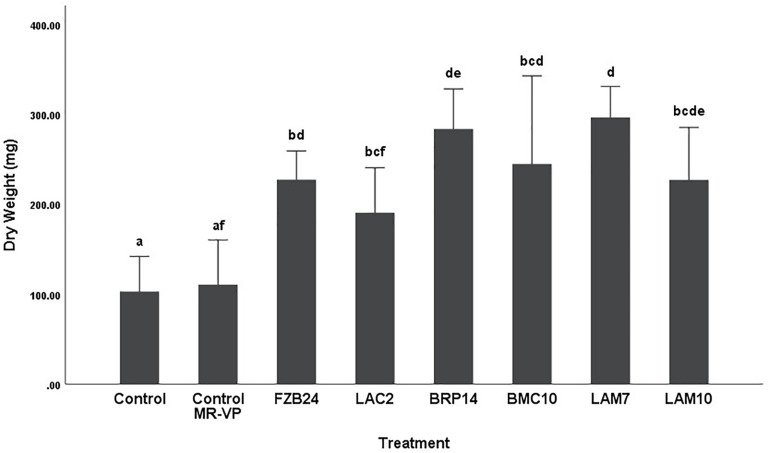
Growth response of plants exposed to BVCs from isolates growing on solid MR-VP media. Dry weight of plants is measured in (mg), error bars represent confidence interval of the mean 99% (*n* = 8). Isolates which share a common letter are not significantly different to one another (*p* > 0.01) according to one-way between groups ANOVA with *post hoc* Tukey test.

**FIGURE 3:**
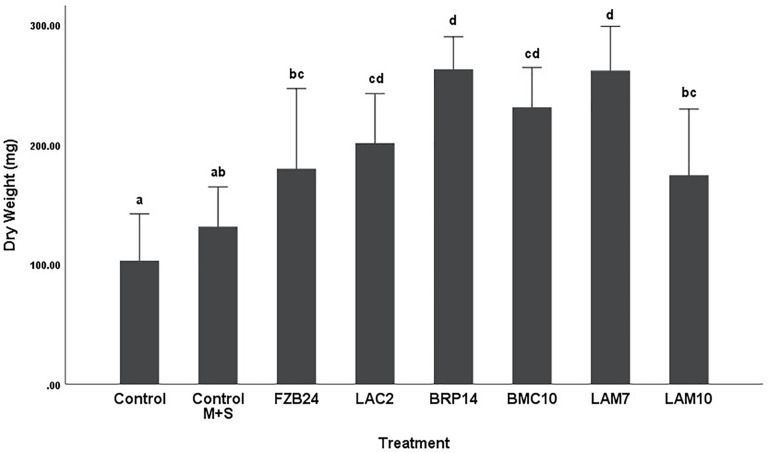
Growth response of plants exposed to BVCs from isolates growing on solid M+S media. Dry weight of plants is measured in (mg), error bars represent confidence interval of the mean 99% (*n* = 8). Isolates which share a common letter are not significantly different to one another (*p* > 0.01) according to one-way between groups ANOVA with *post hoc* Tukey test.

To further examine the main effects of isolate and media on dry weight and stem length, one-way between groups ANOVA was carried out for isolates cultured on both types of media and their effects were determined, respectively. All isolate BVCs increased plant dry weight and stem length compared to plants in both controls when isolates were cultured on MR-VP. When cultured on M + S, all isolate BVCs also increased dry weight compared to both controls and five of six isolates increased stem length compared to both controls. Of the isolates cultured on MR-VP media, BRP14 and LAM7 had the greatest overall effect on biomass increase with a 2.57- and 2.69-fold increase in dry weight compared to that of the control with axenic media treatment respectively (Figure 2). No significant differences in dry weight were observed between respective *Bacillus* isolates (FZB24, BMC10, LAM7) cultured on MR-VP, additionally no significant differences were observed between the two respective *Serratia* isolates (LAC2, LAM10), however LAC2 was the only isolate significantly less effective at promoting plant growth when compared to LAM7 and BRP14 (*p* > 0.01). All isolates growing on M + S media significantly increased dry weight compared to the control treatment (Figure 3). The isolates FZB24 and LAM10 did not display a significant increase in growth compared to the control treatment with axenic M + S media (*p* > 0.01). The remaining isolates LAC2, BMC10, LAM7 and BRP14 significantly increased biomass compared to the axenic media control treatment (*p* < 0.01). The increase in biomass was again most pronounced in plants treated with BRP14 and LAM7 where dry weight was increased by ∼2-fold compared to the control treatment.

**FIGURE 4:**
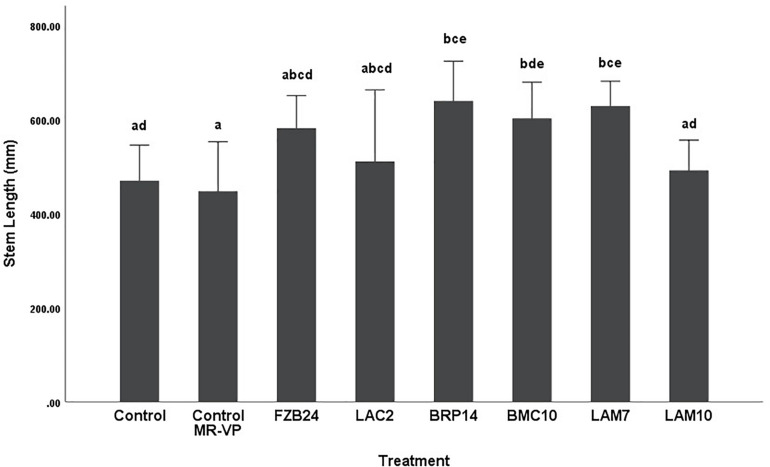
The effect on plant stem length of BVCs from isolates growing on solid MR-VP media. Stem length of plants is measured in (mm), error bars represent confidence interval of the mean (99%) (*n* = 8). Isolates which share a common letter are not significantly different to one another (*p* > 0.01) according to one-way between groups ANOVA with *post hoc* Tukey test.

**FIGURE 5:**
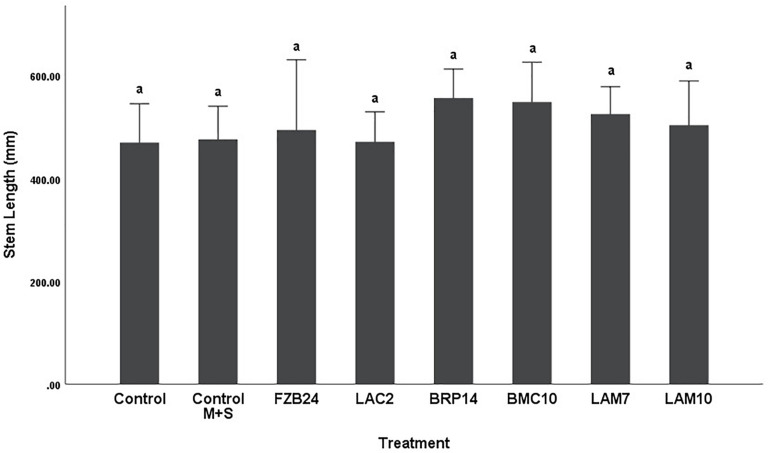
The effect on plant stem length of BVCs from isolates growing on solid M+S media. Stem length of plants is measured in (mm), error bars represent confidence interval of the mean (99%) (*n* = 8). Isolates which share a common letter are not significantly different to one another (*p* > 0.01) according to one-way between groups ANOVA with *post hoc* Tukey test.

Differences in stem length were not as pronounced as dry weight between both media types. In plants treated with MR-VP-derived BVCs, only BRP14 and LAM7 were significantly different from both control treatments (*p* < 0.01). The *Bacillus* isolate BMC10 was also found to significantly increase stem length compared to the axenic control treatment (*p* < 0.01) (Figure 4). No significant differences were observed between the control treatments and the FZB24, LAC2 and LAM10 isolates (*p* > 0.01). The effect of M + S-derived BVCs on stem length was not significantly different between the test isolates and the control treatments (*p* > 0.01) (Figure 5), indicating that media type can affect the rate of growth with respect to stem length.

### Detection of BVCs From Isolates Cultured in Three Different Media Types

Of the 66 BVCs detected across all isolates and media, alcohols were the most abundant (25.75%), followed by ketones (15.15%) and both alkanes and aldehydes (13.63%). Other chemical groups detected include the amines, alkenes, pyrazines and alkynes. All six isolates were capable of producing 2,5-dimethylpyrazine when cultured in TSB. In fact, all three pyrazines listed were only detected when isolates were cultured in TSB and LAC2 was the only isolate to emit each of these three pyrazines with trimethylpyrazine and 3-ethyl-2,5-dimethylpyrazine detected in addition to 2,5-dimethylpyrazine. The production of pyrazines from TSB-derived bacterial cultures has been reported previously (Rees et al., 2017).

Further analysis revealed media type influenced the number and scope of BVCs emitted by isolates. Of the three media types, M + S media was responsible for the least amount of BVCs detected, only 23 BVCs in total were detected in M + S-cultured isolates, with alkanes (30.43%) and ketones (34.78%) being the most prevalent. The best performing isolate in terms of BVC emission potential from M + S was BRP14 which emitted 10 (43.47%) of these 23 BVCs, in contrast, no volatiles of probable biogenic origin were detected when FZB24 was cultured in TSB.

The greatest number of BVC emissions was observed when isolates were cultured in MR-VP with 36 BVCs detected in total, alcohols (36.11%) and alkanes (16.66%) were the most prevalent. The greatest amount of BVCs detected from isolates cultured in MR-VP was from LAC2 which emitted 18 (50%) of the 36 BVCs detected. Unlike the earlier observation in M + S, BRP14 emitted the least amount of BVCs when cultured in MR-VP with only six (16.66%) being detected from this isolate. A total of 32 BVCs were emitted from isolates cultured in TSB with alcohols (31.25%) and ketones (21.87%) being the most prevalent. Similar to the observation in MR-VP, LAC2 emitted the greatest number of BVCs, 15 (46.87%) in total, while BRP14 emitted the least with seven (21.87%) BVCs detected (Table 1).

**TABLE 1:** Putative volatile organic compound production over 72 h from six bacterial isolates growing in tryptic soy broth (TSB), liquid MR-VP and liquid M + S.

Volatile organic compound	TSB	MR-VP	M + S
3-hydroxy-2-butanone	FZB24, BMC10, LAM7, LAM10	FZB24, BMC10, LAM7	BMC10
2,3-butanediol	FZB24, BMC10, LAM7, LAM10	FZB24, BMC10, LAM7	Not Detected
2,5-dimethylpyrazine	FZB24, LAC2, BRP14, BMC10, LAM7, LAM10	Not Detected	Not Detected
3-methyl-1-butanol	LAC2, BRP14, LAM7	LAC2, LAM7	LAC2, LAM7
Dimethylamine	LAC2	LAC2, LAM7	Not Detected
2-butanamine	LAM7	LAC2	Not Detected
1-decanol	LAC2, LAM7	LAC2, LAM7	Not Detected
1-dodecanol	LAC2, LAM7	LAC2, LAM7	Not Detected
2-undecanone	LAC2	LAC2	LAC2
2-tridecanone	LAC2, LAM7	LAC2	LAC2, LAM7
2-heptadecanone	LAC2	LAC2	LAC2
1-methyldecylamine	LAC2	Not Detected	Not Detected
Dodecane	BMC10	FZB24, LAM7	Not Detected
2-piperidinone	Not Detected	LAC2, BMC10, LAM7, FZB24	Not Detected
2-nonanone	LAC2	LAC2	LAC2
1-undecene	BRP14	BRP14	BRP14
Benzaldehyde	FZB24, BMC10, LAM10	BMC10	Not Detected
Dimethyl disulfide	LAM7	Not Detected	Not Detected
2-methyl-1-butanol	Not Detected	LAM7	LAC2
2-ethyl-1-pentanol	Not Detected	LAC2	Not Detected
Hexadecane	BMC10	FZB24, LAC2, LAM7, LAM10	BRP14
2-ethyl-1-hexanol	BMC10, BRP14, LAM10	BMC10, FZB24	Not Detected
2-nonanol	BRP14	Not Detected	Not Detected
1,4-undecadiene	BRP14	Not Detected	Not Detected
2,9-dimethyldecane	Not Detected	BRP14, LAM10	BRP14, LAM10
Decane	Not Detected	BRP14	BMC10
1-octanol	Not Detected	LAM7	Not Detected
1-heptadecanol	LAM7	LAM7, LAC2	Not Detected
Cyclopropyl carbinol	LAC2, BMC10, LAM10	LAC2	Not Detected
1-tetradecanol	Not Detected	LAM7	Not Detected
2-propanamine	LAC2, BMC10	Not Detected	Not Detected
2-octanamine	LAM10	Not Detected	Not Detected
1-tridecanol	Not Detected	LAM7	Not Detected
Undecane	Not Detected	Not Detected	BRP14
Pentanal	Not Detected	LAC2	Not Detected
Octanal	Not Detected	LAC2	Not Detected
Nonanal	Not Detected	BRP14	Not Detected
Decanal	Not Detected	BRP14	Not Detected
3-methylbutanal	Not Detected	Not Detected	LAC2
3-tridecanone	LAC2	Not Detected	Not Detected
4-methyl-2-heptanone	LAM10	Not Detected	BRP14, BMC10
Hexadecanal	Not Detected	LAC2	Not Detected
Di-tert-butyl-dicarbonate	LAM10	Not Detected	BRP14, BMC10, LAM7
5-methylundecane	Not Detected	LAM7, LAC2	BRP14
2-ethyl-4-methyl-1-pentanol	LAC2, LAM10	Not Detected	BMC10, LAM7
Pentanoic Acid	Not Detected	BRP14	BRP14
4-methyldecane, 2,6-dimethylundecane, Ethyne, Ethylene oxide, Cyclobutanol	BMC10	Not Detected	Not Detected
1-nonene, Fluoroethyne, 2-octenal	BRP14	Not Detected	Not Detected
Benzene ethanol, 2-ethynyloxy-ethanol, 1,3-bis(1,1-dimethyl-ethyl)benzene	Not Detected	LAM7	Not Detected
Formic acid	Not Detected	Not Detected	BRP14
4-methyldecane	Not Detected	LAM10	BRP14
Trimethylpyrazine, 3-ethyl-2,5-dimethylpyrazine	LAC2	Not Detected	Not Detected
4-methyl-2-pentanone, 2-ethoxy-2-methylpropane	Not Detected	Not Detected	BMC10, LAM7
4,6-dimethyl-2-heptanone	Not Detected	Not Detected	LAM7
Acetaldehyde	Not Detected	LAM10	Not Detected
2-butynoic acid	Not Detected	Not Detected	LAM10

Unique BVCs were identified in a number of isolate BVC emissions, for example, LAC2 emitted nine BVCs unique to this isolate in our study, among them 2-nonanone, 2-heptadecanone and 2-undecanone which were observed in the emission profiles associated with growth in all three media types. There were 10 unique BVCs emitted by BRP14 and one of these was common to all three media types namely 1-undecene, while other BVCs include 2-nonanol, 1,4-undecadiene, nonanal, decanal, 1-nonene, fluoroethyne, 2-octenal, formic acid and pentanoic acid. BMC10 emitted five unique BVCs; 4-methyldecane, 2,6-dimethylundecane, ethyne, ethylene oxide and cyclobutanol, but only when cultured in TSB. LAM10 was found to emit two unique BVCs; acetaldehyde and 2-butynoic acid, and LAM7 emitted seven unique BVCs; 1-octanol, 1-tetradecanol, tridecanol, benzene ethanol, 2-ethynyloxy-ethanol, 1,3-bis(1,1-dimethyl-ethyl)benzene and dimethyl disulfide, the latter of which has been shown to both promote (Meldau et al., 2013) and slightly inhibit plant growth (Cordovez et al., 2018). A large amount of overlap was observed in the capability of LAC2 and LAM7 to emit the same BVCs as one another namely dimethylamine, 2-butanamine, 1-decanol, 1-dodecanol, 2-tridecanone, 2-methyl-1-butanol and 1-heptadecanol, representing 10.60% of all BVCs detected in our analysis.

### Transcriptomic Response to BVC Exposure

**FIGURE 6:**
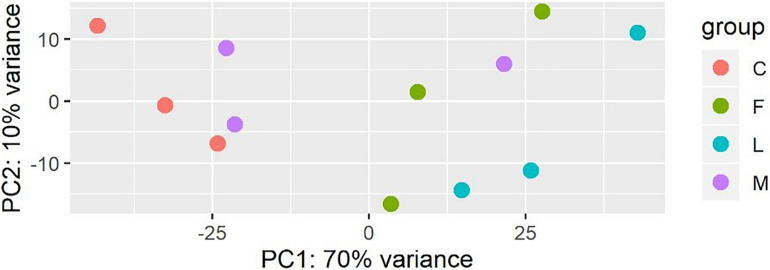
Plot of the first two principal components of a PCA generated from the expression data (count data with variance stabilizing transformation from DeSeq2) colored by condition showing that samples in each condition group together.

**FIGURE 7:**
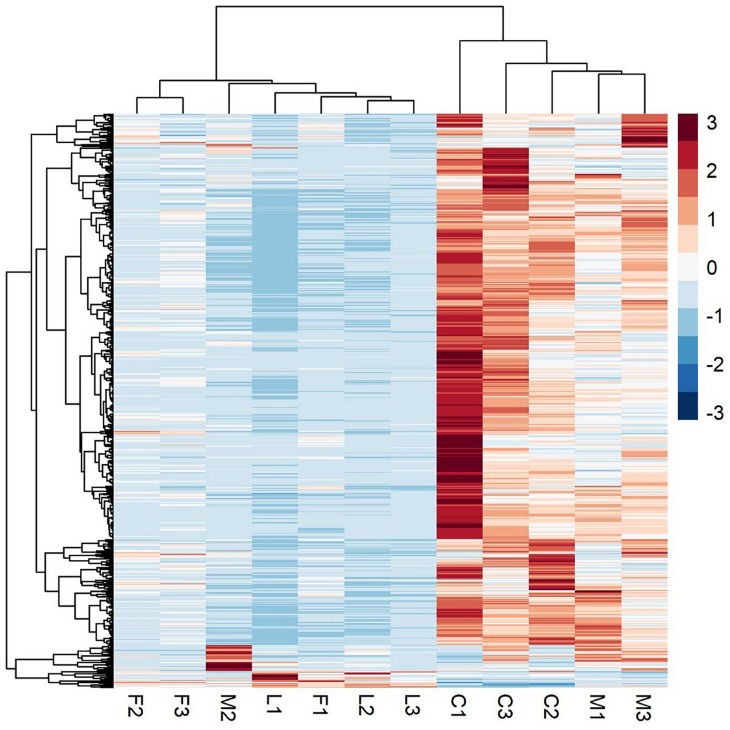
A heatmap of RNA-Seq expression z-scores computed for genes that are significantly differentially expressed Benjamini-Hochberg adjusted *p*-Value (*p* < 0.05) in any comparison. The genes (rows) and samples (columns) are clustered using the Pearson Correlation distance and complete linkage hierarchical clustering. The color code shows the row z-score, with a red color indicating higher expression of a gene and blue color indicating lower expression of a gene. This heatmap demonstrates that C (control) and M (control with axenic MR-VP media) groups, and L (LAC2/MR-VP volatile blend) and F (FZB24/MR-VP volatile blend) groups cluster together and that the genes involved have higher expression in the C and M groups compared with the L and F groups.

A principal component analysis revealed that there were distinct differences in gene expression between BVC treatment and control groups, with treatment accounting for 80% of the variance in gene expression between groups in the first two principal components (Figure 6). A heatmap of differentially expressed genes (DEGs) between control and BVC treatments demonstrates that for the most part the respective treatments co-cluster with one another except for one of the MR-VP axenic media control treatments (M2) (Figure 7). Overall, BVC treatment within our experimental set up, results in the relative downregulation of genes compared to the control treatments, regardless of whether the respective BVC-emitting isolate is from the *Bacillus* or *Serratia* genus. Enrichment of three gene ontologies was observed following plant exposure to BVCs emitted by LAC2, all of which were associated with photosynthesis. Two belong to the biological process, GO:0019684 (photosynthesis, light reaction) and GO:0009772 (photosynthetic electron transport in photosystem II) while the third belonged to molecular function GO:0045156 (electron transporter, transferring electrons within the cyclic electron transport pathway of photosynthesis activity).

**FIGURE 8:**
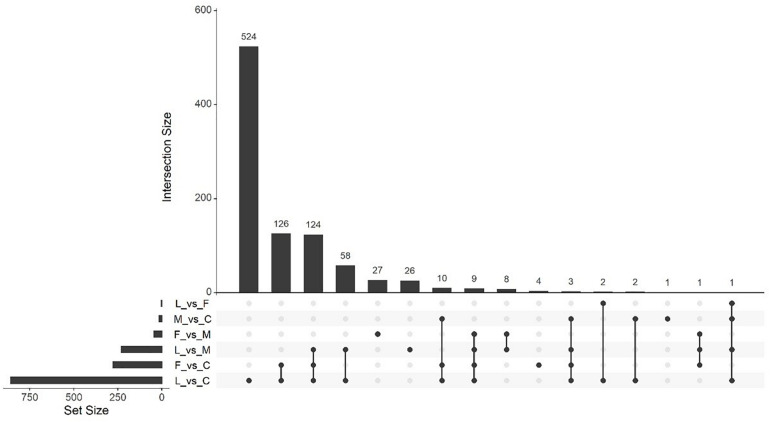
UpSetR plot showing numbers of downregulated differentially expressed genes (DEGs), (*p* < 0.05) and Log2 fold change (≥2.0). Any DEGs common to plant treatments are shown by connected dots. Cumulative numbers of DEGs are calculated by adding numbers associated with dots in each respective treatment row.

Massive analysis of cDNA RNA-seq revealed that in all, 52 genes were upregulated between treatments and, the greatest number of DEGs, 17 altogether, was observed in the (C vs. M) comparison. The LAC2 BVC-treated plants had the next highest levels of upregulated genes with 14 and 13 DEGs observed in the (L vs. C) and (L vs. M) comparisons respectively. Very little upregulation of gene expression was observed in FZB24 BVC-treated plants with the (F vs. C), (F vs. M), and (F vs. L) yielding two, three and three DEGs respectively (Figure 8).

Of the upregulated genes observed in the (L vs. C) comparison a number of genes associated with photosynthetic processes and cell division were observed such as a chlorophyll a/b binding protein and the plastid division regulator MinE along with genes associated with a possible stress and/or defense responses such as Zinc finger (CCCH-type) protein and Hsp20/alpha crystallin family protein. The (L vs. M) comparison also resulted in the upregulation of genes associated with photosynthesis where four chlorophyll a/b binding proteins were identified. In the (F vs. M) comparison the gene for protease inhibitor IIa was the only identifiable transcript from the PGSC genome (Table 2).

**TABLE 2:** Upregulated genes from PGSC genome annotations in response to BVC exposure.

Treatment	Gene ID	Gene name	Log2FC	References
LAC2 vs. Control	PGSC0003DMG400007123	Phytosulfokine peptide	2.075	Igarashi et al., 2012
	PGSC0003DMG400008309	Chlorophyll a/b binding protein	2.097	Zhu et al., 2020
	PGSC0003DMG400022062	Aspartic proteinase	2.413	Resjö et al., 2019
	PGSC0003DMG400028333	Plastid division regulator MinE	2.125	Chen et al., 2018
	PGSC0003DMG400026831	Conserved gene of unknown function	4.159	N/A
	PGSC0003DMG400020916	Fibrillarin homolog	4.499	Rajamäki et al., 2020
	PGSC0003DMG401031741	UPA16	2.274	Kay et al., 2009
	PGSC0003DMG400005792	Nucleoside diphosphate kinase	3.760	Lin et al., 2015
	PGSC0003DMG402025318	Zinc finger (CCCH-type) protein	2.075	Bogamuwa and Jang, 2014
	PGSC0003DMG400033693	UPA16	3.046	Kay et al., 2009
	PGSC0003DMG400008713	Hsp20/alpha crystallin family protein	3.918	Zhao et al., 2018
LAC2 vs. Axenic MR-VP	PGSC0003DMG400000493	Carbonic anhydrase	2.074	DiMario et al., 2018
	PGSC0003DMG400008309	Chlorophyll a/b binding protein	2.289	Zhu et al., 2020
	PGSC0003DMG400005884	Conserved gene of unknown function	3.288	Lekota et al., 2019
	PGSC0003DMG400013416	Chlorophyll a-b binding protein 3C, chloroplastic	2.074	Zhu et al., 2020
	PGSC0003DMG400004301	Chlorophyll a, b binding protein type I	2.185	Zhu et al., 2020
	PGSC0003DMG400007796	DNA-directed RNA polymerase II largest subunit	2.663	Zhou et al., 2017
	PGSC0003DMG400033046	Dof zinc finger protein	2.161	Venkatesh and Park, 2015
	PGSC0003DMG400013412	Chlorophyll a-b binding protein 3C	2.455	Gao and Bradeen, 2016
	PGSC0003DMG400012838	Non-specific lipid-transfer protein	4.402	N/A
	PGSC0003DMG400012839	Non-specific lipid-transfer protein	4.392	N/A
FZB24 vs. Axenic MR-VP	PGSC0003DMG400030593	Proteinase inhibitor IIa	2.088	Rehman et al., 2017

**FIGURE 9:**
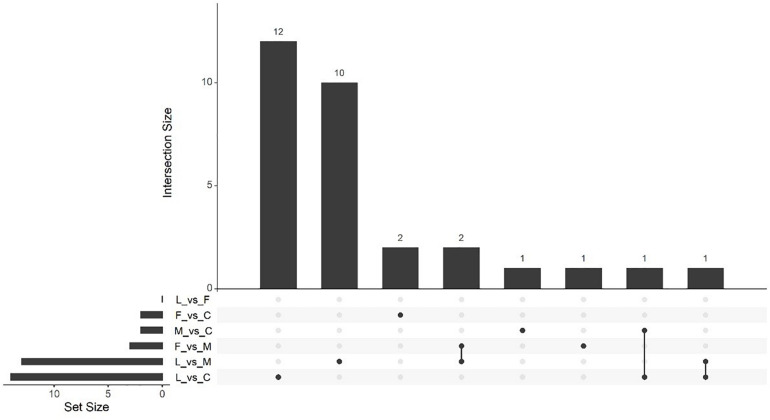
UpSetR plot showing numbers of upregulated differentially expressed genes (DEGs), (*p* < 0.05) and Log2 fold change (≥2.0). Any DEGs common to plant treatments are shown by connected dots. Cumulative numbers of DEGs are calculated by adding numbers associated with dots in each respective treatment row.

Massive analysis of cDNA RNA-seq revealed that 1,413 genes were downregulated across all comparisons, the greatest difference being observed in (L vs. C) with 859 DEGs identified (Table 3). As in the upregulation response, BVC treatment from the LAC2 isolate was responsible for the highest amount of DEG downregulation. The lowest amount of downregulated DEGs was in the (F vs. L) comparison with no DEGs observed (Figure 9). Generally, downregulation was far more pronounced than upregulation in BVC-treated plants with a large proportion of these DEGs being related to defense and stress responses (Table 4).

**TABLE 3:** Numbers of upregulated and downregulated genes between BVC treatments from isolates cultured on MR-VP media according to MACE RNA-seq.

Treatment comparison	Upregulated genes	Downregulated genes
FZB24 vs. Control	2	277
LAC2 vs. Control	14	859
Control vs. Axenic MR-VP	17	2
FZB24 vs. LAC2	3	0
FZB24 vs. Axenic MR-VP	3	45
LAC2 vs. Axenic MR-VP	13	230
Total	52	1,413

**TABLE 4:** Downregulated genes from PGSC genome annotations directly involved in or related to plant defense and stress responses identified post-BVC exposure.

Treatment	Gene ID	Gene name	Log2FC	References
FZB24 vs. Control	PGSC0003DMG400033029	Enhanced disease susceptibility 1 protein	-2.412	El Oirdi and Bouarab, 2007; Venugopal et al., 2009
	PGSC0003DMG400007742	NBS-coding resistance gene protein	-2.190	N/A
	PGSC0003DMG400025437	Stress responsive gene 6 protein, Srg6	-2.043	Zhuang et al., 2012
	PGSC0003DMG400000241	Spotted leaf protein	-2.178	Zeng et al., 2004
	PGSC0003DMG400020587	Rpi-vnt1	-3.014	Roman et al., 2017
	PGSC0003DMG400029220	Tospovirus resistance protein A	-2.464	de Oliveira et al., 2018
	PGSC0003DMG400004413	Tm-2 protein	-2.414	Maayan et al., 2018
	PGSC0003DMG400012025	UPF0497 membrane protein 8	-2.714	Resjö et al., 2019
	PGSC0003DMG400002362	GYF domain-containing protein	-2.321	Wu et al., 2017
LAC2 vs. Control	PGSC0003DMG402030235	Disease resistance protein R3a	-2.570	Engelhardt et al., 2012
	PGSC0003DMG402029058	Disease resistance protein	-3.213	N/A
	PGSC0003DMG400012025	UPF0497 membrane protein 8	-4.407	Resjö et al., 2019
	PGSC0003DMG400021733	TNP2	-2.359	He et al., 2000
	PGSC0003DMG400030239	CC-NBS-LRR protein	-3.895	Goyer et al., 2015
	PGSC0003DMG400017065	Disease resistance protein RGA1	-2.679	Huang et al., 2005
	PGSC0003DMG400030462	Avr9/Cf-9 rapidly elicited protein 216	-3.260	Rowland et al., 2005
	PGSC0003DMG400033029	Enhanced disease susceptibility 1 protein	-3.932	El Oirdi and Bouarab, 2007; Venugopal et al., 2009
	PGSC0003DMG400020099	RPM1 interacting protein 4 transcript 2	-2.052	Lee et al., 2015
	PGSC0003DMG401031196	WRKY transcription factor 16	-2.213	Phukan et al., 2016
	PGSC0003DMG400001550	TSI-1 protein	-2.9046	Dahal et al., 2009
	PGSC0003DMG400028904	SGT1	-2.260	Yu et al., 2019
	PGSC0003DMG400007742	NBS-coding resistance gene protein	-3.812	N/A
	PGSC0003DMG400032555	NAC domain protein	-2.174	Kwenda et al., 2016
	PGSC0003DMG400004053	Protein CASP	-2.641	N/A
	PGSC0003DMG400021331	PEN1	-2.231	Collins et al., 2003; Nielsen et al., 2012
	PGSC0003DMG400016769	Double WRKY type transfactor (StWRKY8)	-2.163	Gao et al., 2020
	PGSC0003DMG400009238	Leucine-rich repeat family protein	-2.109	N/A
	PGSC0003DMG402029995	TMV resistance protein N	-3.358	Qian et al., 2018
	PGSC0003DMG400031839	RING-H2 finger protein ATL2	-2.898	Serrano and Guzmán, 2004
	PGSC0003DMG400009634	Bacterial spot disease resistance protein 4	-3.018	Schornack et al., 2004
	PGSC0003DMG400025437	Stress responsive gene 6 protein, Srg6	-2.531	Malatrasi et al., 2002
	PGSC0003DMG400024365	Rpi-vnt1	-4.841	Roman et al., 2017
	PGSC0003DMG400019104	Heat shock protein 70 (HSP70)-interacting protein	-2.261	Jiang et al., 2014
	PGSC0003DMG400000241	Spotted leaf protein	-2.903	Zeng et al., 2004
	PGSC0003DMG400044116	Beta-glucan-binding protein 4	-2.599	Fliegmann et al., 2004
	PGSC0003DMG400004327	Leucine-rich repeat resistance protein	-2.123	N/A
	PGSC0003DMG404026432	Tir-nbs-lrr resistance protein	-4.939	Li et al., 2017
	PGSC0003DMG401011899	Tospovirus resistance protein B	-2.391	de Oliveira et al., 2018
	PGSC0003DMG400029220	Tospovirus resistance protein A	-3.154	De Oliveira et al., 2016
	PGSC0003DMG400019667	Potato resistance I2GA-SH23-3	-2.709	Huang et al., 2005
	PGSC0003DMG401030700	Resistance gene	-3.319	Gu et al., 2020
	PGSC0003DMG400029371	DNA-binding protein NtWRKY3	-2.209	Liu et al., 2004b
	PGSC0003DMG400047046	Nematode resistance	-2.675	Goyer et al., 2015
	PGSC0003DMG400047346	BRASSINOSTEROID INSENSITIVE 1-associated receptor kinase 1	-2.153	Bentham et al., 2020
	PGSC0003DMG402026149	Mitogen-activated protein kinase kinase kinase	-2.646	Pitzschke et al., 2009
LAC2 vs. Axenic MR-VP	PGSC0003DMG400030462	Avr9/Cf-9 rapidly elicited protein 216	-2.871	Rowland et al., 2005
	PGSC0003DMG400020099	RPM1 interacting protein 4 transcript 2	-2.547	Lee et al., 2015
	PGSC0003DMG400033029	Enhanced disease susceptibility 1 protein	-2.758	Venugopal et al., 2009
	PGSC0003DMG400019232	GRAS2	-2.851	Chang et al., 2015
	PGSC0003DMG400019824	JA-induced WRKY protein	-2.030	Wiesel et al., 2015
	PGSC0003DMG401031196	WRKY transcription factor 16	-2.644	Phukan et al., 2016
	PGSC0003DMG400028904	SGT1	-2.472	Yu et al., 2019
	PGSC0003DMG400007742	NBS-coding resistance gene protein	-2.564	N/A
	PGSC0003DMG400021331	PEN1	-2.248	Collins et al., 2003; Nielsen et al., 2012
	PGSC0003DMG401030700	Resistance gene	-2.550	Gu et al., 2020
	PGSC0003DMG400002562	F-box/kelch-repeat protein	-2.366	Wang et al., 2017
FZB24 vs. Axenic MR-VP	PGSC0003DMG400002562	F-box/kelch-repeat protein	-2.417	Wang et al., 2017

## Discussion

For almost two decades the interactions of BVCs with plants has been the focus of intense research. These interactions can be directly observed in the sensor plant, usually through the induction of a defense response via the upregulation of pathogenesis-related (PR) genes which are key for induced systemic resistance (ISR) or through an increased rate in plant growth and accumulation of biomass (Ryu et al., 2004; Kim et al., 2015). BVCs can also have indirect effects on plants via the inhibition of bacterial and especially fungal phytopathogens (Giorgio et al., 2015) and have also been observed to increase the rate of micronutrient-uptake in sensor plants, leading to a potentially biofortified harvest in crop plants (Orozco-Mosqueda et al., 2013).

We devised a novel growth system in which to study the effects of BVCs on potato plants in an enclosed, passively ventilated experimental setup. The model plant *Arabidopsis thaliana* is often the predominant target in lab-based *in vitro* studies of the BVC-mediated effects on plants (Bee Park et al., 2013; Sharifi and Ryu, 2016; Bhattacharyya et al., 2015). Here, we present the growth effects of BVCs on potato to study their effects on a major staple food crop plant. The type of growth system, either open or closed, in which to observe the effect of microbial VOCs on plants, phytopathogens and animals has been widely researched and discussed in recent years (Kai et al., 2007; Popova et al., 2014; Park et al., 2015; Kai et al., 2016; Sharifi and Ryu, 2018; Song et al., 2019). Extrapolating how these respective systems affect volatile concentrations, mixtures of volatiles, responses of test organisms and the combined spatiotemporal effects of these parameters has made the definitive translation of effects from the lab setting to the field more difficult. The physical nature of VOCs, such as a high vapor pressure, low boiling point and low molecular weight (<300 Da) lends to their short-term proximity to target organisms in the field, although a number of methodologies have been developed to mitigate this effect (Song and Ryu, 2013; Fincheira et al., 2019, 2020).

This study identified VOCs from six bacterial isolates from three genera; *Bacillus*, *Serratia*, and *Pseudomonas* cultured in three different types of liquid media via SPME-GC/MS. The growth medium of the bacteria is known to alter the blend of BVCs which are emitted (Blom et al., 2011a) and taking this into account we selected TSB, MR-VP and M + S liquid media from which to assess both the BVC-producing capability and variety from selected isolates. We identified 66 different putative BVCs in total, some of which are widely reported in the literature such as 2,3-butanediol, 3-hydroxy-2-butanone, 2,5-dimethylpyrazine, 2-nonanone, 2-tridecanone and 3-methyl-1-butanol (Ryu et al., 2003; Velivelli et al., 2015; Asari et al., 2016; Fincheira et al., 2017; Fincheira and Quiroz, 2018; Fincheira and Quiroz, 2019). Some BVCs were also observed to be isolate-specific such as 1-undecene, 2-nonanone and 1-tetradecanol produced by BRP14, LAC2 and LAM7 respectively. Interestingly, dimethyl disulfide, widely reported as a PGP volatile among many PGP bacterial isolates (Meldau et al., 2013; Tyc et al., 2017) was only detected once in our analysis by a single strain, LAM7, and only when cultured in TSB. Dimethyl disulfide has also been reported as a growth inhibitor with concentration-dependent effects (Kai et al., 2010; Cordovez et al., 2018).

When isolates cultured on MR-VP agar were co-cultivated with potato microplants there was a significant increase in growth between the control and all BVC treatments. This may be attributed to the carbon-rich composition of this media and the subsequent blend and/or concentration of BVCs present within the growth system. The most common BVCs observed between isolates utilizing MR-VP as a carbon source were hexadecane and 2-piperidinone, with four of six isolates emitting these particular BVCs, the latter was recently described as an insecticidal constituent from a biocontrol fertilizer (Qiu et al., 2019). The next most common were 3-hydroxy-2-butanone and 2,3-butanediol identified in the headspace of three isolates, FZB24, BMC10 and LAM7 all of which are members of *Bacillus*. The largest increases in dry biomass were observed after exposure to BVCs from LAM7 and BRP14, 2.69- and 2.57-fold greater than that of the axenic media control respectively, suggesting that the volatile blends from these isolates cultured in MR-VP are strong growth-promoters. Our analysis identified 2,3-butanediol and 3-hydroxy-2-butanone within the headspace of *B. mycoides* (LAM7), interestingly these were not detected in the headspace of *B. mycoides* strains (CHT2401) and (CHT2402) in a previous study (Huang et al., 2018). This may indicate that bacterial strains within a particular species may harbor the ability to employ strain-specific BVCs for exploitation of particular ecological niches under the correct environmental conditions as described for *Bacillus subtilis* (Kai, 2020), additionally, interlaboratory variation in volatile profiles cannot be discounted (Clark et al., 2021). Stem length was also significantly increased after exposure to BVCs from both LAM7 and BRP14 in comparison to both control treatments. There was a total of 18 volatiles identified in the LAM7/MR-VP BVC blend and consisted of, amongst others, 3-methyl-1-butanol, 1-decanol, 1-dodecanol, hexadecane, 1-octanol and 1-tridecanol which were also identified as growth promoters in previous studies (Velázquez-Becerra et al., 2011; Bee Park et al., 2013; Velivelli et al., 2015; Pérez-Flores et al., 2017; Camarena-Pozos et al., 2019). In BRP 14/MR-VP the predominant BVC, from a total blend of just five, was identified as 1-undecene, a particularly potent volatile in the biocontrol effect against some fungal phytopathogens mediated by many *Pseudomonas* strains (Kong et al., 2020). The greatest effect on stem length was observed with plants exposed to BVCs of BRP14/MR-VP.

A similar trend was seen in dry weight measurements from M + S-derived BVC treatments as was observed in MR-VP, however LAC2 BVCs marginally increased dry weight compared to those of FZB24 when cultured on M + S. No significant differences in stem length were observed post-exposure to M + S-cultured isolates to that of the respective control treatments. These differences in growth observed between both media types can be explained by the composition of the respective media, with MR-VP being a more carbon-rich media than M + S. Indeed, we observed that proliferation of isolates across the media surface was far more apparent in isolates which were cultured on MR-VP, while isolates cultured on M + S rarely expanded their radial growth beyond the point at which their starting culture was dropped on to the media. Members of the genus *Serratia* are reported as BVC-emitting biocontrol and PGP agents (Wenke et al., 2019) and while their BVC-mediated inhibition of phytopathogenic growth has been well reported, the effect of volatiles from two *Serratia* strains in this study, LAC2 and LAM10, were often not as effective as those of *Bacillus* and *Pseudomonas* at increasing plant growth. LAC2 was the only isolate observed to produce 2-undecanone, 2-heptadecanone and 2-nonanone which have been the subject of particular research focus in recent years, particularly 2-nonanone both in the preservation of commercially important fruits such as strawberry from *Botrytis cinerea* (Abarca et al., 2017), as an inhibitor of bacterial quorum sensing, a disruptor of protein folding in bacteria and inducer of plant defense (Chernin et al., 2011; Melkina et al., 2017; Báez-Vallejo et al., 2020).

For transcriptomic analysis of potato plants exposed to BVCs in our system the isolates LAC2 and the commercially available isolate FZB24, members of *Serratia* and *Bacillus* respectively were chosen as the BVC source due to the fact they only share two common BVCs when grown on MR-VP, namely 2-piperidinone and hexadecane. Additionally, species of these genus have been well documented to emit antimicrobial and growth-promoting BVCs (Fincheira et al., 2016; Tahir et al., 2017). Therefore, we expected to see noticeable alterations in the plant transcriptomic landscape in response to these isolates. Interestingly, only 52 genes were upregulated across treatments, whereas 1,413 were downregulated. In BVC-treated plants, LAC2 was responsible for the upregulation of genes involved in photosynthetic activity, such as a number of genes coding for chlorophyll a/b binding proteins and the plastid division regulator MinE. Genes associated with defense and stress responses were also upregulated such as phytosulfokine peptide, aspartic proteinase and zinc finger (CCCH-type) protein. FZB24 was responsible for the upregulation of just one identifiable gene from the PGSC potato genome, proteinase inhibitor IIa, which is known to be involved in the defense response against phytopathogens and insect pests (Rehman et al., 2017; Table 2). The gene phytosulfokine peptide has been reported as an attenuator of pattern-triggered immunity in *Arabidopsis* and acts as a positive regulator of growth by balancing resource allocation between defense and growth (Igarashi et al., 2012). Unexpectedly, many of the downregulated genes were related to defense and stress responses. A similar response has been reported previously from the pure PGP volatile 1-decene emitted by the beneficial fungus *Trichoderma* which downregulated stress and defense genes such as WRKY transcription factors (Lee et al., 2019). Three WRKY transcriptions factors were also downregulated in our study. Five DEGs related to stress and/or defense were commonly downregulated in (L vs. C) and (F vs. C) namely enhanced disease susceptibility 1 protein (EDS1); stress responsive gene 6 protein, Srg6; Rpi-vnt1; tospovirus resistance protein A and UPF0497 membrane protein 8. The protein (EDS1) has been shown, in association with salicylic acid (SA), to mediate redundant functions in R protein-mediated defense signaling confirmed by simultaneous mutations of EDS1 and a salicylic acid-synthesizing enzyme named SID2 resulting in a compromised defense and/or hypersensitive response mediated by R proteins which contain a coiled-coil domain residing in their N-terminal domains (Venugopal et al., 2009). A membrane protein involved in cell wall strengthening, UPF0497, was shown to be upregulated in the PTI response to *Phytophthora infestans* (Resjö et al., 2019), this class of membrane protein was also downregulated in our study. The fact that there are only three BVCs shared in common between LAC2 and FZB24 suggests that these BVCs may be responsible for the induction of the five commonly downregulated DEGs between these isolates and is worthy of future investigation.

Three DEGs downregulated in our analysis code for proteins named PEN1, EDS1 and SGT1 which are known to play crucial roles in plant defense, especially non-host resistance (NHR) to fungal pathogens. These three genes work in a hierarchical manner and sometimes directly co-operate to mediate the hosts innate defense response. PEN1 (PENETRATION 1) is involved in diminishing the successful entry of phytopathogens into the plant cell. *PEN1* encodes a syntaxin anchored in the plasma membrane which is part of the SNARE (soluble *N*-ethylmaleimide-sensitive factor attachment protein receptor) complex and is required for the timely arrival of papillae which contain callose and extracellular membrane material that aids penetration resistance (Collins et al., 2003; Nielsen et al., 2012). As part of the SNARE complex, the PEN1 protein forms part of the activation of pre-invasion defense in the resistance to powdery mildews in Arabidopsis (Lipka et al., 2008). In the event that the frontline defense of the PEN1/SNARE complex fails, mechanisms to deal with post-invasion defense come into play, where EDS1 and SGT1 play a significant role. The post-invasion NHR response requires EDS1, phytoalexin-deficient 4 (PAD4) and senescence-associated gene 101 (SAG101) and has been confirmed through mutational analyses (Lipka et al., 2005; Stein et al., 2006). These proteins are homologous to lipases and comprise a regulatory node that is crucial for basal defense against the oomycete *Hyaloperonospora parasitica*, SA-mediated signaling and some resistance pathways mediated by R-genes (Kwon et al., 2008). As part of a crucial NHR response EDS1 is necessary for the resistance, including the hypersensitive response, mediated by TIR (Toll/interleukin 1 receptor)-containing proteins (Hu et al., 2005; Wiermer et al., 2005). SGT1 is also essential for the correct functioning of several R genes and the post-invasion NHR response in Arabidopsis and is a highly conserved constituent required for the function of certain Skp1/Cullin/F-Box protein (SCF)-type E3 ubiquitin ligase complexes (Austin et al., 2002; Liu et al., 2004a; Holt et al., 2005; Azevedo et al., 2006; Yu et al., 2019). While EDS1 and SGT1 have been shown to be essential components of the plant innate immune system, the never-ending arms race between pathogen and host can turn these genes into clandestine pathogenic weapons. El Oirdi and Bouarab (2007) demonstrated that *B. cinerea* can essentially flank the defenses of the Solanaeceous member *Nicotiana benthamiana* using a molecular distraction. EDS1 and SGT1 are part of the control mechanism for SA-dependent disease resistance toward biotrophic pathogens, while resistance against necrotrophic pathogens is mediated through the jasmonic acid (JA) pathway (McDowell and Dangl, 2000; da Cunha et al., 2006). The SA and JA pathways are known to be antagonistic (Mur et al., 2006). EDS1 acts as an activator of SA signaling but as a repressor of JA/Ethylene signaling (Brodersen et al., 2006), therefore the authors concluded that the activation of EDS1 and SGT1 signaling dampened the JA signaling response thus allowing *B. cinerea* pathogenesis to progress through exploitation of the plants own innate immune system (El Oirdi and Bouarab, 2007).

A number of research questions arising from the use of this growth system require further investigation. Most notably that of the effect of inorganic volatiles such as CO_2_ and NH_3_ emitted from bacterial cultures which can have both positive and negative effects on plant growth respectively (Kai and Piechulla, 2009; Weise et al., 2013; Piechulla et al., 2017). Gas exchange between the internal and external environment was not monitored in this study and likely plays an important role in the levels of growth and differential gene expression in the plants within this system due to different plant species, bacteria, culture media, filter types, temperature and photoperiod. A number of methodologies could be employed to further investigate this such as CO_2_ trapping (Kai and Piechulla, 2009), alteration of Microbox^®^ HEPA filter porosity with accompanying trace gas analysis of CO_2_ for example and determination of the effect of differing concentrations of pure inorganic, and indeed organic, volatiles such as NH_3_ and 2R, 3R-butanediol (Fincheira et al., 2021). Additionally, the effect of bacterial cell number to levels of volatile emissions within the system needs to be determined as this is known to ultimately effect plant growth (Kai, 2020). The use of liquid rather than solid media within the system may also affect BVC emission profiles as oxygen availability between both physical states of media can lead to shifts in volatile emissions (Farag et al., 2006; Somerville and Proctor, 2013; Kai et al., 2020). It is important to note that liquid media was agitated in our experimental procedure for putative VOC detection and this should be considered in any future comparative analyses. Additionally, opening and closing the lid of the system to exchange liquid media for example, may affect any internal equilibrium of volatiles and increase the risk of phytopathogenic contamination.

The use of this passively ventilated system has proved viable for observing the effect of BVCs on plant growth, and may mitigate the effects observed in closed growth systems which often lack sufficient space for many plants other than *Arabidopsis thaliana* seedlings to grow freely. In many *in vitro* BVC-plant studies, fold changes in plant growth promotion can be quite striking (Blom et al., 2011a; Park et al., 2015). In our study the growth promotion is more modest, perhaps owing to the passive ventilation of the system, which may more closely mimic BVC kinetics found in open natural environments. The methodologies of Park et al. (2015); Cordovez et al. (2018) nicely demonstrate active exposure of volatiles from live bacterial cultures, however experimental conditions in these studies either result in the plant being exposed to the open air environment or enclosed in containers with no ventilation to the outer atmosphere, depending on whether BVC exposure was directed to the roots or shoots. Our system allows exposure of the shoots and roots to BVCs, while BVCs may first interact with the shoots, the co-cultivation time of 4 weeks allows ample opportunity for BVC diffusion through PE foam to the roots. Comparative transcriptomic analyses between the root systems of control vs. BVC-exposed plants should yield answers to this question. Furthermore, while sterilized soil may be lacking live microbes with potential PGP activity, it will still retain organic matter which can as a nutrient source thus masking or inadvertently misrepresenting, the absolute effect of BVCs on PGP potential, this is an implication which deserves further study. It should be noted that in our study root proliferation throughout the foam made it too difficult to obtain an adequate root length and root dry weight as it was not possible to simply wash away or melt the foam, as can be achieved using soil or agar, from the roots, while this may complicate accurate measurements of root parameters described above, it should not prohibit transcriptomic analyses of roots. The observed differences in gene expression between the stems of control vs. BVC-exposed plants within our system shows that foliar application of the PGPR tested in this system could possibly yield similar results in the greenhouse and/or field, this remains to be seen. The observation that genes involved in attenuating and mediating the plant immune response, both upregulated and downregulated respectively, demonstrates that BVC-mediated communication with the plant can involve more than just the upregulation of defense genes. Rather, the induction of a careful dance between up- and downregulation of a suite of genes, perhaps to optimize the pre-colonization internal environment of the host for an endophytic symbiont. If the bacterial symbiont has an epiphytic life strategy in the rhizosphere of the host plant, and depends on host-derived root exudates as a carbon source, this dance may maintain the overall health status of the host by dampening the negative physiological effects of an over-reactive defense response. The use of this system is quite affordable and may allow for the study of more cumbersome or older plants that cannot be studied in relatively restrictive I-plates. Future analysis on the effect of plant type, plant substrate, microbe, microbial growth media, filter porosity and other parameters relevant to BVC-plant interactions in this system may help to elucidate specific genes or pathways activated in response to a particular single BVC or blend. The opportunity also presents itself to analyze the tripartite effect of fungal/bacterial/plant volatile organic compound interactions within this experimental setup. Systems such as this can help elucidate genes and/or their pathways modulated via BVC exposure for a better understanding of their effect on plant health and ultimately for better solutions to the looming concern of global food security in an ecologically uncertain future.

## Data Availability Statement

Datasets for plant growth are available from the corresponding author. Isolate 16S rRNA sequence data has been deposited in GenBank under accession numbers MT373405, MT373394, MT373410, MT373402, and MT373403. RNA-seq data has been deposited in NCBI’s GEO Series under accession number GSE160297.

## Author Contributions

DH-D and BD devised the experimental setup and layout. DH-D carried out the growth experiments, GC/MS analysis, and growth data analysis and compiled the main text. DH-D and ED extracted total RNA from plant tissue and carried out quality control of RNA and shipping to GenXPro. SC carried out all bioinformatic analyses of MACE RNA-seq data. All authors contributed to the proofreading and editing of the manuscript.

## Conflict of Interest

The authors declare that the research was conducted in the absence of any commercial or financial relationships that could be construed as a potential conflict of interest.

## Footnotes

1 https://github.com/marcelm/cutadapt/
